# Variable Is Better Than Invariable: Sparse VSS-NLMS Algorithms with Application to Adaptive MIMO Channel Estimation

**DOI:** 10.1155/2014/274897

**Published:** 2014-06-24

**Authors:** Guan Gui, Zhang-xin Chen, Li Xu, Qun Wan, Jiyan Huang, Fumiyuki Adachi

**Affiliations:** ^1^Department of Electronics and Information Systems, Akita Prefectural University, Akita 015-0055, Japan; ^2^Department of Electronic Engineering, University of Electronic Science and Technology of China, Chengdu 611731, China; ^3^Department of Communications Engineering, Tohoku University, Sendai 980-8579, Japan

## Abstract

Channel estimation problem is one of the key technical issues in sparse frequency-selective fading multiple-input multiple-output (MIMO) communication systems using orthogonal frequency division multiplexing (OFDM) scheme. To estimate sparse MIMO channels, sparse* invariable step-size normalized least mean square *(ISS-NLMS) algorithms were applied to adaptive sparse channel estimation (ACSE). It is well known that step-size is a critical parameter which controls three aspects: algorithm stability, estimation performance, and computational cost. However, traditional methods are vulnerable to cause estimation performance loss because ISS cannot balance the three aspects simultaneously. In this paper, we propose two stable* sparse variable step-size *NLMS (VSS-NLMS) algorithms to improve the accuracy of MIMO channel estimators. First, ASCE is formulated in MIMO-OFDM systems. Second, different sparse penalties are introduced to VSS-NLMS algorithm for ASCE. In addition, difference between sparse ISS-NLMS algorithms and sparse VSS-NLMS ones is explained and their lower bounds are also derived. At last, to verify the effectiveness of the proposed algorithms for ASCE, several selected simulation results are shown to prove that the proposed sparse VSS-NLMS algorithms can achieve better estimation performance than the conventional methods via mean square error (MSE) and bit error rate (BER) metrics.

## 1. Introduction

**Figure 1:**
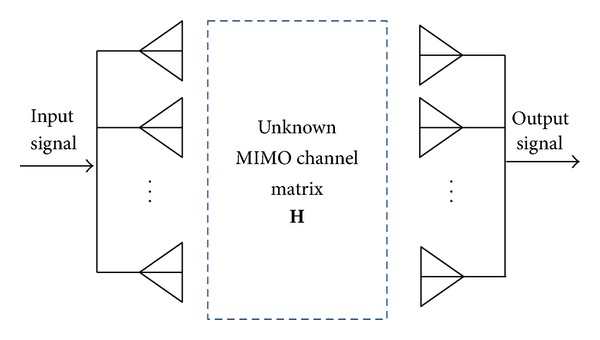
Signal transmission over MIMO channel.

High-rate data broadband transmission over multiple-input multiple-output (MIMO) channel has become one of the mainstream techniques for the next generation communication systems [1]. The major motivation is due to the fact that MIMO technology, as shown in Figure 1, is a way of using multiple antennas to simultaneously transmit multiple streams of data in wireless communications [2] and hence it can bring considerable improvements such as data rate, reliability, and energy efficiency. In fact, coherent receivers require accurate channel state information (CSI) since the received signals are distorted by multipath fading transmission. The accurate estimation of channel impulse response (CIR) is a crucial aspect and challenging issue in coherent modulation and its accuracy has a significant impact on the overall performance of the communication system.

**Figure 2:**
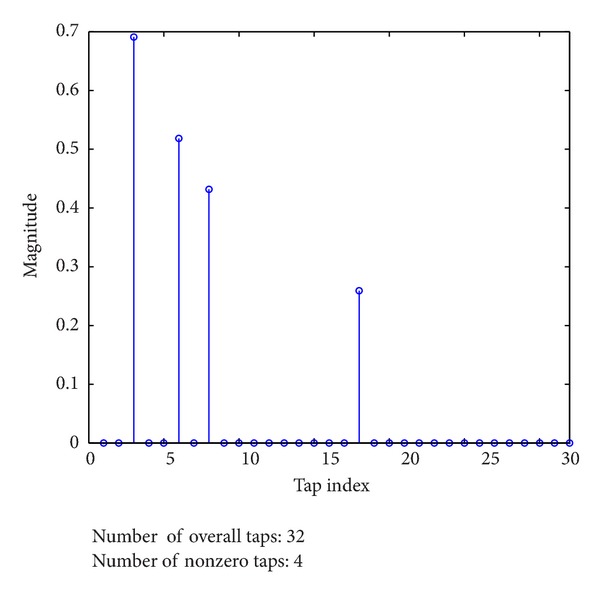
A typical example of sparse multipath channel.

During last decades, there exist many channel estimation methods which were proposed for MIMO systems [3–11]. All these methods are categorized into two groups. The first group contains the linear channel estimation methods, for example, least squares (LS) algorithm, based on the assumption of dense CIRs. By applying these approaches, the performance of linear methods depends only on the size of MIMO channel. Note that narrowband MIMO channel may be modeled as dense channel model because of its very short time delay spread. Accurately, broadband MIMO channel is often modeled as sparse channel model [12–14]. A typical example of sparse channel is shown in Figure 2. It is well known that linear channel estimation methods are relatively simple to implement due to their low computational complexity [3–8]. Unfortunately, their main drawback is the failure to exploit the inherent channel sparsity. The second group is the sparse channel estimation methods which use compressive sensing (CS) theory [15, 16]. Interested authors are recommended to refer to [17]. Basically, optimal sparse channel estimation often requires that its training signal satisfies restrictive isometry property (RIP) [18] in high probability. However, designing the RIP-satisfied training signal is a nonpolynomial (NP) hard problem [19]. Also, there exist some proposed methods which are stable with the cost of extra computational burden, especially in time-variant MIMO systems. For example, sparse channel estimation method using Dantzig selector was proposed for double-selective fading MIMO systems [10]. Indeed, the proposed method needs to be solved by linear programming which incurs high computational complexity. To reduce the computational cost, sparse channel estimation methods using greedy iterative algorithms were also proposed in [9, 11]. But their complexity still depends on the number of nonzero taps of MIMO channel.

Unfortunately, the mentioned proposed methods do not have adaptive estimation capability. Adaptive sparse channel estimation (ASCE) methods using sparse* invariable step-size* (ISS) least mean square algorithms (ISS-LMS) were proposed in [20] for single-input single-output (SISO) channels. However, conventional ISS-LMS methods have two main drawbacks: (1) sensitive to random scale of training signal and (2) unstable in low signal-to-noise ratio (SNR) regime.

To overcome the two harmful factors on channel estimation and extend their applications to estimate MIMO channels, sparse ISS normalized least mean square (ISS-NLMS) algorithms, for example, zero-attracting ISS-NLMS (ZA-ISS-NLMS) and reweight ZA-ISS-NLMS (RZA-ISS-NLMS), were proposed in [21]. It is well known that* step-size* is a critical parameter which controls the estimation performance, convergence rate, and computational cost. Different from conventional sparse ISS-NLMS algorithms [21],* zero-attracting variable step-size NLMS* (ZA-VSS-NLMS) algorithm was proposed for ASCE to improve estimation performance in sparse multipath single-input single-output (SISO) systems [22]. Unlike the previous works, this paper proposes two sparse VSS-NLMS algorithms for estimating sparse MIMO channels. The main contribution of this paper is summarized as follows. First, we derive the lower bound of proposed MIMO channel estimator for introducing the research motivation. Second, we extend the proposed VSS-ZA-NLMS for estimating SISO channels in [22] to MIMO channels. Third, a reweighted ZA-VSS-NLMS (RZA-VSS-NLMS) is proposed to further improve the estimation performance of MIMO channels. In addition, we explain the reason why sparse VSS-NLMS algorithms can achieve better performance than conventional sparse ISS-NLMS ones. Finally, Monte Carlo based computer simulations are conducted to confirm the effectiveness of our proposed algorithms via two metrics: bit error rate (BER) and mean square error (MSE).

The remainder of this paper is organized as follows. A baseband MIMO system model is described and problem formulation is presented in Section 2. In Section 3, sparse ISS-NLMS algorithms are overviewed. In Section 4, sparse VSS-NLMS algorithms are proposed and a figure example is also given to explain the difference between ISS and VSS based algorithms. Simulation results are presented in Section 5 in order to assess the proposed methods. Finally, we conclude the paper in Section 6.

*Notations.* Capital bold letters and small bold letters denote matrices and row/column vectors, respectively. The discrete FOURIER transform (DFT) matrix is denoted by **F** with entries [**F**]_*kn*_ = 1/*K* **e** ^−*j*2*πkq*/*K*^, *k*, *q* = 0,1,…, *K* − 1; (·)^*T*^, (·)^*H*^, (·)^−1^and |·| denote the transpose, conjugate transpose, matrix inversion, and absolute operations, respectively; *E*{·} denotes the expectation operator; assume any vector **h** = [*h*_0_,…,*h*_*l*_,…,*h*_*L*−1_  ]^*T*^; ||**h**||_1_ and ||**h**||_2_ denote *l*_1_-norm, that is, ||**h**||_1_ = ∑_*l*_|*h*_*l*_|, and *l*_2_-norm, that is, ||**h**||_2_ = (∑_*l*_|*h*_*l*_|^2^)^1/2^; sgn⁡(**h**) is a component-wise function which is defined as sgn⁡(*h*) = 1 for *h* > 0, sgn⁡(*h*) = 0 for *h* = 0, and sgn⁡(*h*) = 1 for *h* < 0, where *h* denotes any component in vector **h**; $\tilde{h}$ represents the channel estimator of **h**.

## 2. System Model

**Figure 3:**
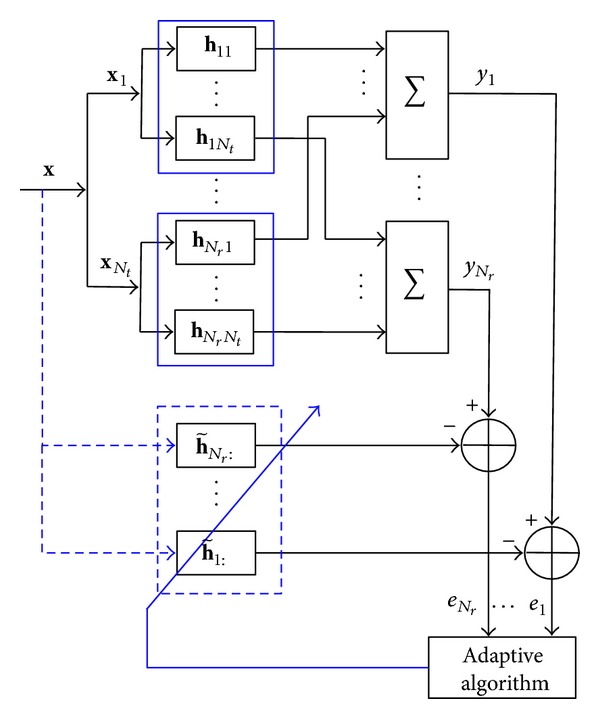
Adaptive algorithm for estimating MIMO channels.

**Figure 4:**
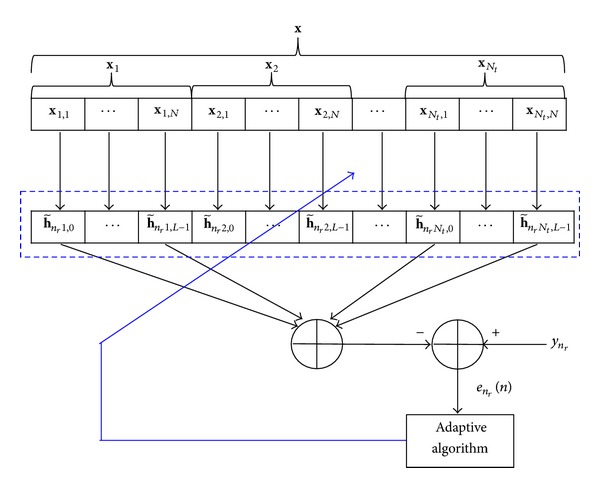
MISO channel estimation at *n*_*r*_th antenna of the receiver.

A frequency-selective fading MIMO communication system using OFDM modulation scheme is considered in Figure 3. Initially, frequency domain signal vector ${\overset{-}{x}}_{n_{t}}\left(t\right)={\left[{\overset{-}{x}}_{n_{t}}\left(t,0\right),\ldots ,{\overset{-}{x}}_{n_{t}}\left(t,K-1\right)\right]}^{T}$, *n*_*t*_ = 1,2,…, *N*_*t*_, is fed to inverse discrete Fourier transform (IDFT) at the *n*_*t*_th antenna, where *K* is the number of subcarriers and *N*_*t*_ is the number of transmit antennas. Assume that the transmit power is normalized as $E\left\{{||{\overset{-}{x}}_{n_{t}}\left(t\right)||}_{2}^{2}\right\}=1$. The resultant vector $x_{n_{t}}\left(t\right)≜F^{H}{\overset{-}{x}}_{n_{t}}\left(t\right)$ is padded with cyclic prefix (CP) of length *L*_CP_ ≥ (*K* − 1) to avoid interblock interference (IBI). After CP removal, the received signal vector at the *n*_*r*_th antenna for time *t* is written as *y*_*n*_*r*__(*t*), where *n*_*r*_ = 1,2,…, *N*_*r*_. As shown in Figure 4, the received signal vector **y** and input signal vector **x**(*t*) are related by

(1) ynr(t)=∑nt=1NthnrntTxnt(t)+znr(t)=hnr:Tx(t)+znr(t),

where **x**(*t*) = [**x**_1_^*T*^(*t*),**x**_2_^*T*^(*t*),…,**x**_*N*_*t*__^*T*^(*t*)]^*T*^ collects all of the input signal vectors from different antennas at the transmitter, *z*_*n*_*r*__(*t*) is an additive white Gaussian noise (AWGN) variable with distribution *CN*(0, *σ*_*n*_^2^), and *n*_*r*_th received multiple-input single-output (MISO) channel vector **h**_*n*_*r*_:_ is written as

(2) hnr:∶=[hnr1,0,…,hnr1,L−1︸hnr1T,…,hnrnt,0,…,hnrnt,L−1︸hnrntT,…,kkkkhnrNt,0,…,hnrNt,L−1︸hnrNtT]T

and the matrix-vector form of system model (1) is also written as

(3) $$\begin{array}{c} y=Hx+z, \end{array}$$

where received signal vector **y**, noise vector **z**, and channel matrix **H** can be represented, respectively, as follows:

(4) $$\begin{array}{c} y={\left[y_{1}(t),y_{2}(t),\ldots ,y_{N_{r}}(t)\right]}^{T}\in C^{N_{r}\times 1},\\ z={\left[z_{1}(t),z_{2}(t),\ldots ,z_{N_{r}}(t)\right]}^{T}\in C^{N_{r}\times 1},\\ H=\left[\begin{array}{c} h_{1:}^{T}\\ h_{2:}^{T}\\ \vdots \\ h_{N_{r}:}^{T} \end{array}\right]=\left[\begin{array}{cccc} h_{11}^{T} & h_{12}^{T} & \cdots & h_{1N_{t}}^{T}\\ h_{21}^{T} & h_{22}^{T} & \cdots & h_{2N_{t}}^{T}\\ \vdots & \vdots & \ddots & \vdots \\ h_{N_{r}1}^{T} & h_{N_{r}2}^{T} & \cdots & h_{N_{r}N_{t}}^{T} \end{array}\right]\in C^{N_{r}\times N_{t}L}, \end{array}$$

where **h**_*n*_*r*_*n*_*t*__, *n*_*r*_ = 1,2,…, *N*_*t*_, is assumed to be equal *L*-length sparse channel vector from receiver to *n*_*t*_th antenna. In addition, we also assume that each channel vector **h**_*n*_*r*_*n*_*t*__ is only supported by *T* dominant channel taps.

## 3. Overview of Sparse ISS-NLMS Algorithms

According to the system model in (1), the *n*_*t*_th updating estimation error *e*_*n*_*r*__(*n*) can be written as

(5) enr(n)=ynr(t)−ynr(n)=ynr(t)−h~nr:T(n)x(t),

where ${\tilde{h}}_{n_{r}:}(n)$ denotes an MISO channel estimator of the **h**_*n*_*r*_:_; **e**(*n*) = [*e*_1_(*n*),*e*_1_(*n*),…,*e*_*N*_*r*__(*n*)]^*T*^ denotes receive error vector at the *n*th adaptive update; and *y*_*n*_*r*__(*t*) is the received signal at the *n*_*r*_th receive antenna.

### 3.1. ISS-ZA-NLMS

According to (5), the cost function of ISS-ZA-LMS [23] at the *n*_*r*_th antenna of the receiver can be constructed as

(6) $$\begin{array}{c} G_{\text{ZA},n_{r}}\left(n\right)=\frac{1}{2}e_{n_{r}}^{2}\left(n\right)+\lambda_{\text{ZA}}{||{\tilde{h}}_{n_{r}:}\left(n\right)||}_{1}, \end{array}$$

where *λ*_ZA_ is a regularization parameter to balance the square estimation error *e*_*n*_*r*__^2^(*n*) and sparse penalty of ${\tilde{h}}_{n_{r}:}(n)$. Hence, the corresponding update equation of ISS-ZA-LMS [23] for MIMO channel estimation is derived as

(7) h~nr:(n+1)=h~nr:(n)−μ∂GZA,nr(n)∂h~nr:(n)=h~nr:(n)+μenrx(t)−γZAsgn⁡(h~nr:(n)),

for *n*_*r*_ = 1,2,…, *N*_*r*_, where *γ*_ZA_ = *μλ*_ZA_ and *μ* is the ISS. To mitigate random scaling of input signal **x**(*t*), based on the ISS-ZA-LMS algorithm in (7), the update equation of improved ISS-ZA-NLMS [20, 23] was proposed as

(8) $$\begin{array}{c} {\tilde{h}}_{n_{r}:}\left(n+1\right)={\tilde{h}}_{n_{r}:}\left(n\right)+\frac{\mu e_{n_{r}}x\left(t\right)}{x^{T}\left(t\right)x\left(t\right)}-\gamma_{\text{ZA}}\mathrm{sgn}\left({\tilde{h}}_{n_{r}:}\left(n\right)\right). \end{array}$$

### 3.2. ISS-RZA-NLMS

It is well known that ISS-ZA-LMS cannot distinguish between zero taps and nonzero taps as it gives the same penalty to all the taps which are often forced to be zero with the same probability; therefore, its performance will degrade in less sparse systems. Motivated by the reweighted *l*_1_-norm minimization recovery algorithm [24], Chen et al. have proposed a heuristic approach to reinforce the zero attractor which was termed as the ISS-RZA-LMS [25]. The cost function of ISS-RZA-LMS is given by

(9) GRZA,nr(n)  =12enr2(n) +λRZA∑nt=1Nt∑l=0L−1log⁡(1+εRZA|h~nrnt,l(n)|),

where *λ*_RZA_ > 0 is the regularization parameter and reweighted factor *ε*_RZA_ > 0 is the positive threshold. In computer simulation, the threshold is set as *ε*_RZA_ = 20 which is also suggested in previous papers [26, 27]. The *l*th coefficient ${\tilde{h}}_{n_{r},l}(n)$ of ISS-RZA-LMS channel estimator ${\tilde{h}}_{n_{r}}(n)$ is then updated as

(10) h~nrnt,l(n+1)=h~nrnt,l(n)−μ∂GRZA,nr∂h~nrnt,i(n)=h~nrnt,l(n)+μenrx(t−l) −γRZAsgn⁡(h~nrnt,l(n))1+εRZA|h~nrnt,l(n)|,

for *n*_*r*_ = 1,2,…, *N*_*r*_, where *γ*_RZA_ = *μλ*_RZA_ *ε*_RZA_. According to (10), hence, ISS-RZA-NLMS [20, 23] was proposed as

(11) h~nr:(n+1)=h~nr:(n)+μenrx(t)xT(t)x(t) −γRZAsgn⁡(h~nr:(n))1+εRZA|h~nr:(n)|.

Note that the sparse penalty term $\mathrm{sgn}({\tilde{h}}_{n_{r}:}(n))/(1+\varepsilon_{\text{RZA}}\left|{\tilde{h}}_{n_{r}:}(n)\right|)$ in (11) replaces these channel coefficients $\left\{{\tilde{h}}_{n_{r}n_{t},l}\left(n\right),l=0,1,\ldots ,L-1,n_{t}=1,2,\ldots ,N_{t}\right\}$ under the threshold 1/*ε*_RZA_ as zero.

### 3.3. Drawback of the Sparse ISS-LMS Algorithms

Comparing the standard ISS-NLMS algorithm [28], sparse ISS-NLMS algorithms have a common ability of exploiting channel sparsity. Without the loss of generality, we derive the steady-state mean square error (MSE) performance of the ISS-ZA-NLMS [23] as for the typical example to illustrate the drawbacks of the sparse ISS-NLMS algorithms. Assuming that ${\tilde{H}}_{\text{ISS}}\left(n\right)$ denotes the sparse MIMO channel estimator, under the independence assumption, in [25], the steady-state MSE of ISS-ZA-NLMS estimator ${\tilde{H}}_{\text{ISS}}\left(n\right)$ was derived as

(12) ΔISS(∞)=lim⁡n→∞E{||(H~ISS(n)−H)x(t)||22}=lim⁡n→∞E{∑nr=1Nr||(h~nr:(n)−h)x(t)||22}=Tr⁡[R(I−μR)−1]σn2Nr2−Tr⁡[R(I−μR)−1] +γ1ρZA(ρZA−2γ2/γ1)(2−Tr⁡[R(I−μR)−1])μ≤Tr⁡[R(I−μR)−1]σn2Nr2−Tr⁡[R(I−μR)−1]≤λmax⁡σn2Nr2−3μλmax⁡,

where $\gamma_{1}=E\left[\mathrm{sgn}\left({\tilde{h}}_{n_{r}:}\left(n\right)\right){\left(I-\mu R\right)}^{-1}\mathrm{sgn}\left({\tilde{h}}_{n_{r}:}\left(n\right)\right)\right]>0$ and $\gamma_{2}=E{||{\tilde{h}}_{n_{r}:}\left(\infty \right)||}_{1}-{||h_{n_{r}:}||}_{1}$. To exploit the channel sparsity, *ρ*_ZA_ should be selected in the range (0, 2*γ*_2_/*γ*_1_] so that (*ρ*_ZA_ − 2*γ*_2_/*γ*_1_) ≤ 0. According to (12), the lower bound of Δ_ISS_(*∞*) depends on the three factors: {*λ*_max⁡_, *σ*_*n*_^2^, *μ*}. However, *λ*_max⁡_ and *σ*_*n*_^2^ are determined by the input signal **x**(*t*) and additive noise *z*(*t*), respectively. Only selecting the smaller step-size can further achieve better MSE performance. However, if small step-size *μ* is adopted, it will incur slow convergence speed (i.e., high computation complexity) on overall adaptive channel estimation. Hence, it is expected that large step-size is used in the case of large MSE to accelerate the convergence speed, while small step-size is used in the case of smaller MSE to improve the steady-state MSE performance. Assume $\tilde{H}(n)$ denotes the *n*th update MIMO channel estimator using sparse VSS-NLMS algorithms. As *μ* → 0, the lower bound of steady-state MSE of sparse VSS-NLMS algorithms is derived as

(13) ΔVSS(∞)=lim⁡n→∞E{||(H~(n)−H)x(t)||22}=lim⁡n→∞E{∑nr=1Nr||(h~nr:(n)−h)x(t)||22}≤lim⁡μ→0λmax⁡σn2Nr2−3μλmax⁡=λmax⁡σn2Nr2≤ΔISS(∞).

To simultaneously achieve higher convergence speed and lower steady-state MSE performance, we propose sparse VSS-NLMS algorithms for estimating MIMO channels in the next section.

## 4. Proposed Sparse VSS-NLMS Algorithms for Estimating MIMO Channels

Recall that the ISS-ZA-NLMS algorithm in (8) does not make use of the VSS rather than ISS. Inspirited from the VSS-NLMS algorithm which has been proposed in [29], to improve estimation performance of MIMO channels, sparse VSS-NLMS algorithms are proposed. Unlike the sparse ISS-NLMS algorithm, sparse VSS-NLMS algorithms are time-variant with respect to the accuracy of updating estimators.

### 4.1. VSS-ZA-NLMS

At time *t*, based on the previous research on the ISS-ZA-NLMS and VSS-NLMS algorithms, VSS-ZA-NLMS algorithm is proposed as follows:

(14) h~nr(n+1)=h~nr(n)+μnr(n)eZA(n)x(t)xT(t)x(t) −γZAsgn⁡(h~nr(n)),

where *μ*_*n*_*r*__(*n*) is the VSS which is given by

(15) $$\begin{array}{c} \mu_{n_{r}}\left(n\right)=\mu_{\max}\cdot \frac{p_{n_{r}}^{T}\left(n\right)p_{n_{r}}\left(n\right)}{p_{n_{r}}^{T}\left(n\right)p_{n_{r}}\left(n\right)+C}, \end{array}$$

where *C* is a positive threshold parameter which is related to received signal-to-noise ratio (SNR), *C* ~ *O*(1/SNR). According to (15), the range of VSS is given as *μ*_*n*_*r*__(*n*) ∈ (0, *μ*_max⁡_), where *μ*_max⁡_ is the maximal step-size of gradient descent. Theoretically, the maximal step-size is less than 2 to ensure the adaptive algorithm stability [28]. Please note that **p**_*n*_*r*__(*n*) in (15) is given by

(16) $$\begin{array}{c} p_{n_{r}}\left(n\right)=\beta p_{n_{r}}\left(n-1\right)+\left(1-\beta \right)\frac{x\left(t\right)e_{n_{r}}\left(n\right)}{x^{T}\left(t\right)x\left(t\right)}, \end{array}$$

where *β* ∈ [0,1) is a smoothing factor to trade off VSS and estimation error.

### 4.2. VSS-RZA-NLMS

The *l*th channel coefficient ${\tilde{h}}_{n_{r},l}\left(n\right)$ of ${\tilde{h}}_{n_{r}}\left(n\right)$ is then updated by

(17) h~nr,l(n+1)=h~nr,l(n)+μ(n)enr(n)x(t)xT(t)x(t) −γRZAsgn⁡(h~nr,l(n))1+εRZA|h~nr,l(n)|.

Then, the matrix-vector form of (15) can also be expressed as

(18) h~nr(n+1)=h~nr(n)+μ(n)enr(n)x(t)xT(t)x(t) −γRZAsgn⁡(h~nr(n))  1+εRZA|h~nr(n)|.

Please note that the second term in (16) attracts the channel coefficients ${\tilde{h}}_{n_{r},l}\left(n\right),\;l=0,1,\ldots ,L-1$, whose magnitudes are comparable to 1/*ε*_RZA_ to zeros. For estimating MIMO channels, two proposed sparse VSS-NLMS algorithms are summarized in Algorithm 1.

**Remark 1 .**

**Figure 5:**
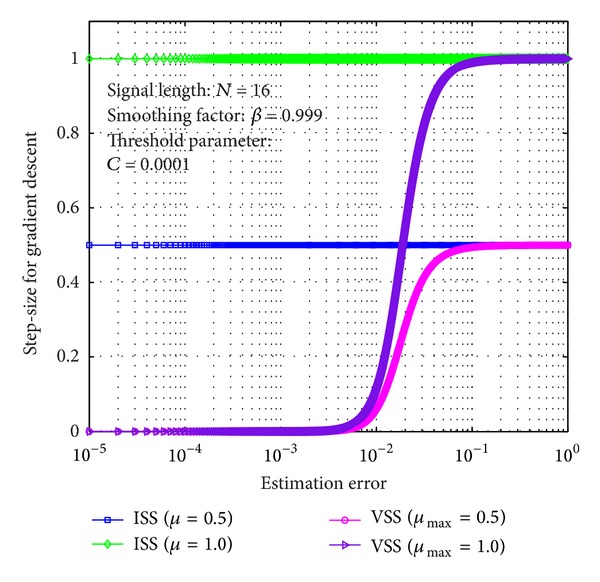
ISS and VSS versus updating estimation error.

To better understand the difference between ISS and VSS, based on (8), (11), and (15), it is worth mentioning that step-size *μ* for sparse ISS-NLMS algorithm is invariable but the step-size *μ*_ZA_(*n*) for sparse VSS-NLMS algorithm is variable as depicted in Figure 5, where the maximal step-size and ISS are set as *μ*_max⁡_ ∈ {0.5,1.0} and *μ* ∈ {0.5,1.0}, respectively. From the figure, one can easily find that ISS is kept invariant. Unlike the ISS, VSS *μ*(*n*) decreases adaptively as the estimation performance increases and vice versa. In other words, sparse VSS-NLMS algorithms adopting VSS for adaptive gradient descend; large step-size is adopted to speed up convergence rate for reducing computational complexity; small step-size is adopted to ensure algorithm stability in the case of high-accuracy estimator for further improving estimation performance.

## 5. Computer Simulations

To confirm the effectiveness of the proposed methods, two metrics, that is, MSE and BER, are adopted for performance evaluation. Channel estimators are evaluated by average MSE which is defined by

(19) $$\begin{array}{c} \text{Average}\;\text{MSE}\left\{\tilde{H}\left(n\right)\right\}=E\left\{{||H-\tilde{H}\left(n\right)||}_{2}^{2}\right\}, \end{array}$$

and system performance is evaluated by the BER metric which adopts different data modulation schemes, such as phase shift keying (PSK) and quadrature amplitude modulation (QAM). The results are averaged over 1000 independent Monte-Carlo runs. The length of each channel vector {**h**_*n*_*r*_*n*_*t*__, *n*_*r*_ = 1,2,…, *N*_*r*_, *n*_*t*_ = 1,2,…, *N*_*t*_} is set as equal length with *L* = 16 and corresponding number of dominant taps is set to *T* ∈ {1,4}. Each dominant channel tap follows random Gaussian distribution as *CN*(0, *σ*_**h**_^2^) and their positions are randomly decided within the length of **h**_*n*_*r*_*n*_*t*__. In addition, MISO channel vector **h**_*n*_*r*_:_ is subject to *E*{||**h**_*n*_*r*_:_||_2_^2^} = 1. The received SNR is defined as *P*_0_/*σ*_*n*_^2^, where *P*_0_ is the power of received signal. Computer simulation parameters are listed in Table 1. Based on the research work in [30], it is worth mentioning that threshold parameters of sparse VSS-NLMS algorithms are adopted *C* = 10^−4^ for 5 dB and *C* = 10^−5^ for 10 dB and 20 dB, respectively.

**Figure 6:**
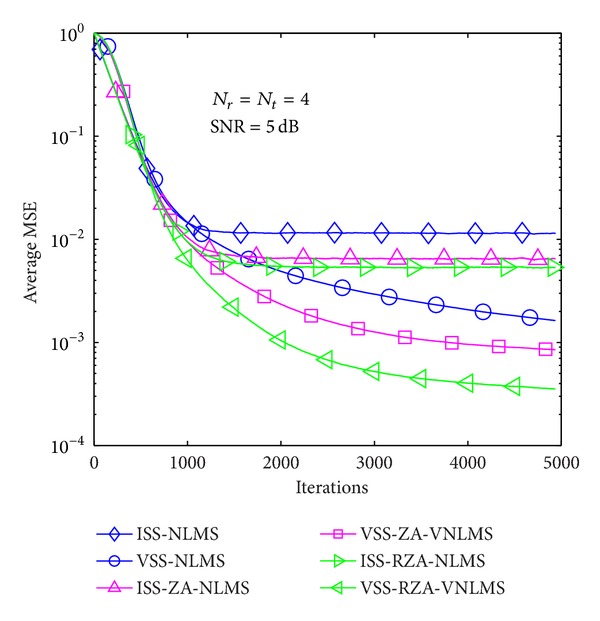
Average MSE performance versus received iterations (*T* = 1).

**Figure 7:**
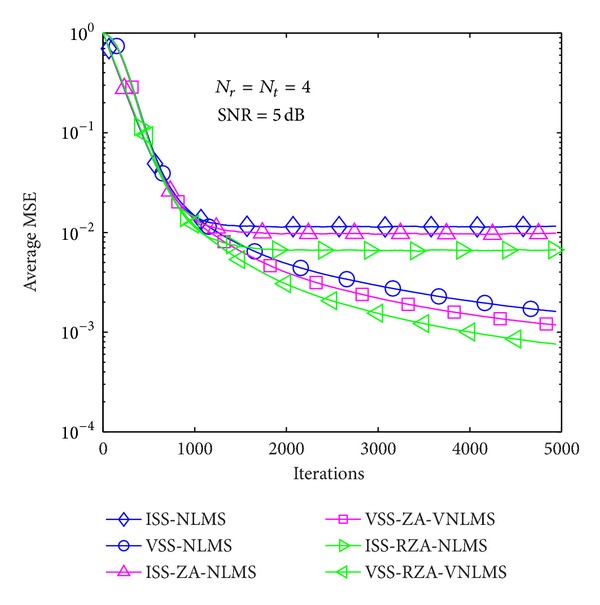
Average MSE performance versus received iterations (*T* = 4).

**Figure 8:**
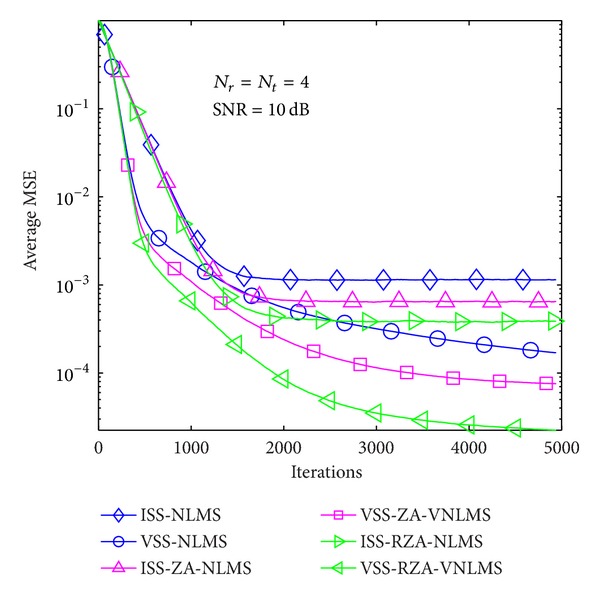
Average MSE performance versus received iterations (*T* = 1).

**Figure 9:**
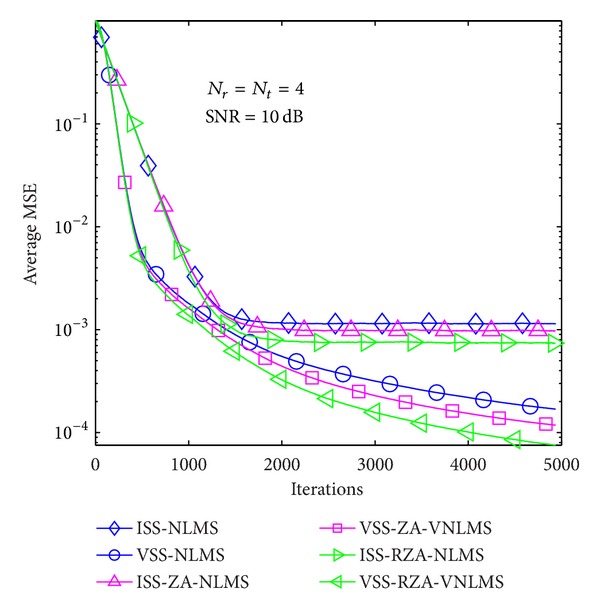
Average MSE performance versus received iterations (*T* = 4).

**Figure 10:**
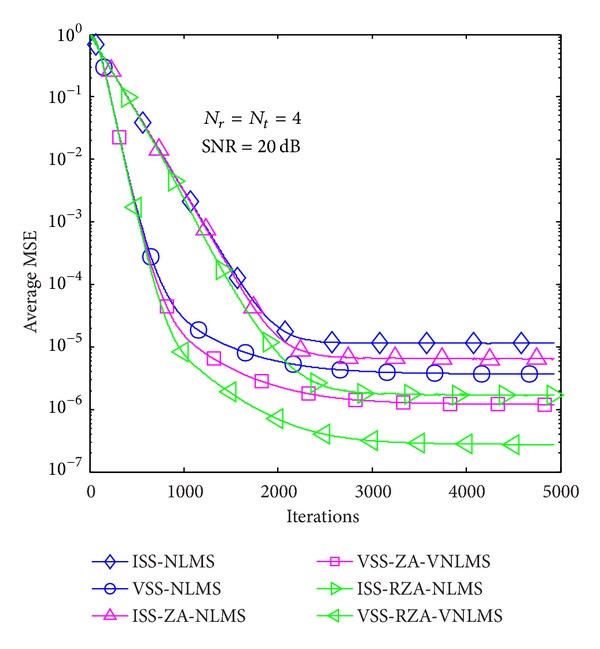
Average MSE performance versus received iterations (*T* = 1).

**Figure 11:**
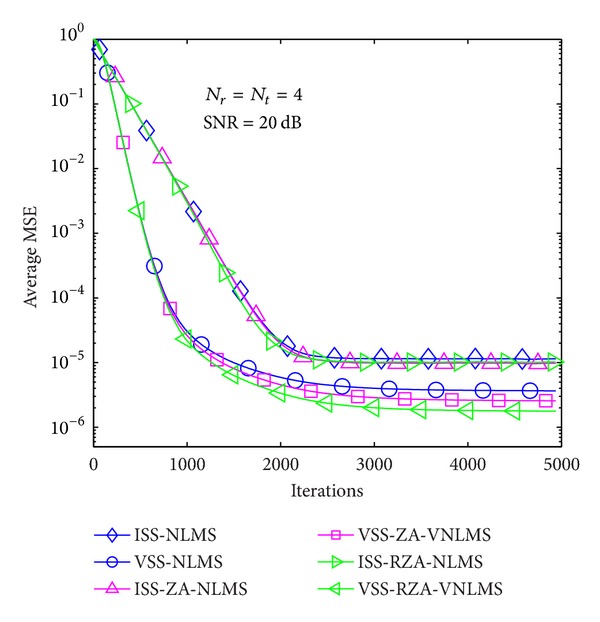
Average MSE performance versus received iterations (*T* = 4).

In the first example, average MSE performance of proposed methods is evaluated in the case of *T* = 1 and 4 in Figures 6, 7, 8, 9, 10, and 11 under three SNR regimes, that is, 5 dB, 10 dB, and 20 dB. To confirm the effectiveness of the proposed method, we compare it with previous methods, that is, ISS-NLMS [28], VSS-NLMS [29], and sparse ISS-NLMS [23, 25]. In addition, to achieve a better steady-state estimation performance, regularization parameters for sparse VSS-NLMS algorithms, that is, VSS-ZA-NLMS and VSS-RZA-NLMS, are adopted from [27], which depend on the number of nonzero taps of a channel. In the case of different SNR regimes, for example, 5 dB, 10 dB, and 20 dB, as shown in Figures 6, 7, 8, 9, 10, and 11, two proposed methods achieved better estimation performance than sparse ISS-NLMS ones.

As it can be observed from Figures 5 and 6, since sparse VSS-NLMS algorithms take advantage of the channel sparsity as for prior information, hence they achieve better estimation performance than standard VSS-NLMS algorithm, especially in a very sparse channel case, for example, *T* = 1. The sparse VSS-NLMS algorithms can exploit much more sparse information for sparser channel. It is obviously observed that the performance gaps between proposed methods and previous methods in Figure 6 (*T* = 1) are bigger than the gaps in Figure 7 (*T* = 4).

**Figure 12:**
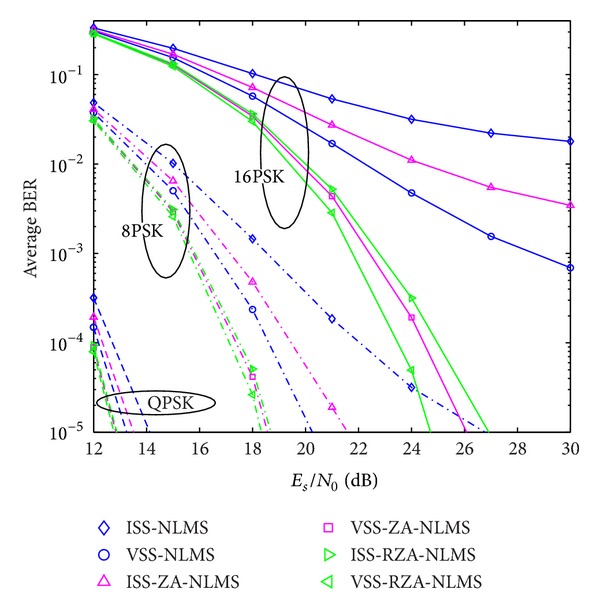
Average BER performance versus received SNR (PSK).

**Figure 13:**
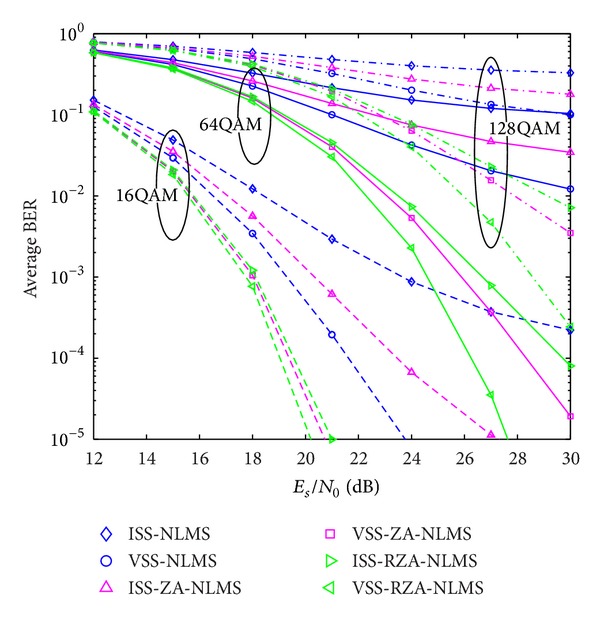
Average BER performance versus received SNR (QAM).

**Figure 14:**
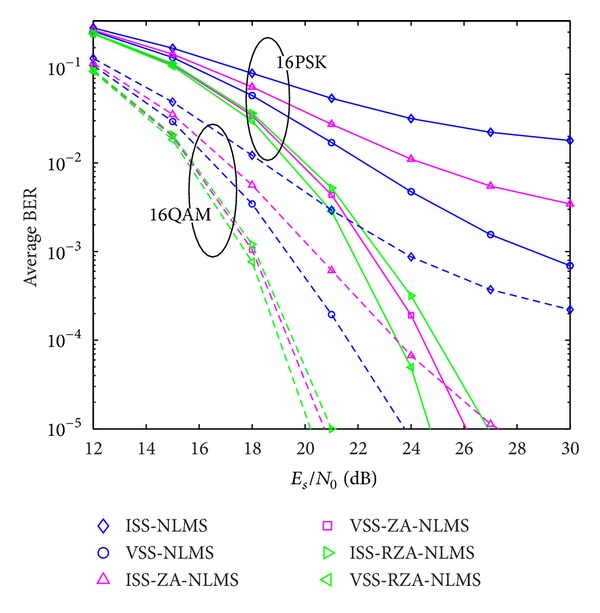
Comparison between 16PSK and 16QAM modulation schemes.

In the second example, system performance using proposed channel estimators is also evaluated with respect to BER performance. Two kinds of signal modulation schemes, that is, multiple PSK and multiple QAM, are considered. Received SNR is defined by *E*_0_/*N*_0_, where *E*_0_ is the average received power of symbol and *N*_0_ is the noise power. In Figure 12, multiple PSK schemes, that is, QPSK, 8PSK, and 16PSK, are considered for data modulation and system performance was evaluated. One can find that there is no big performance difference using QPSK and 8PSK due to high transmission. If the higher-order data modulation is adopted, much better BER performance will be achieved when compared with previous methods, that is, ISS-NLMS, VSS-NLMS, and sparse ISS-NLMS algorithms. In Figure 13, multiple QAM schemes, that is, 16QAM, 64QAM, and 128QAM, are considered for data modulation. One can easily find that the proposed method can achieve a better estimation than previous methods. In addition, we also compare the system performance with respect to different modulation schemes, PSK and QAM. In Figure 14, 16PSK and 16QAM are adopted as for a typical example of performance evaluation. We can find that 16QAM based system performance is better than 16PSK based system using the same channel estimators.

## 6. Conclusion

Traditional adaptive MIMO channel estimation methods utilize sparse ISS-NLMS algorithms using ISS. One of the main disadvantages of the traditional methods is the inability to balance the convergence speed and the estimation accuracy. In this paper, two sparse VSS-NLMS algorithms were proposed for estimating MIMO channels. Unlike the traditional sparse ISS-NLMS algorithms, the proposed algorithms utilized VSS which can change adaptively as the estimation error. Simulation results were provided to confirm the effectiveness of the proposed methods in three aspects: convergence speed, estimation performance, and system performance. First, convergence speed of the proposed methods using VSS is faster than ISS-NLMS based methods due to the fact that VSS for adaptive gradient descent is more efficient than ISS. In other words, VSS can well balance that fast convergence speed is dominant in the case of large estimation error while high accuracy is dominant in the case of small estimation error. Second, the proposed adaptive estimators can achieve better MSE gain than previous methods in different SNR regimes especially for sparser channels. At last, system performance using the proposed channel estimators can also achieve better BER performance than previous methods especially in high-order modulation signal based systems.

## Figures and Tables

**Algorithm 1:**
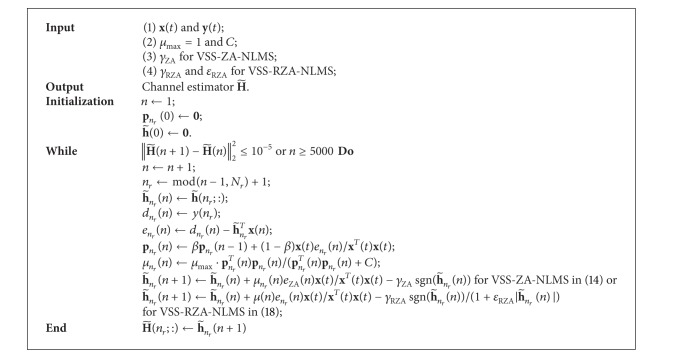
Sparse VSS-NLMS algorithms for estimating MIMO channels.

**Table 1:** Simulation parameters.

Parameters	Values
Number of transceivers (*N*_*t*_, *N*_*r*_)	(4,4)

Channel length of each **h**_*n*_*r*_*n*_*t*__	*L* = 16

Number of nonzero coefficients	*T* ∈ {1,4}

Distribution of nonzero coefficient	Random Gaussian *CN*(0,1)

Threshold parameter for VSS-NLMS	*C* ∈ {10^−4^, 10^−5^}

Received SNR for channel estimation	{5 dB, 15 dB, 25 dB}

Received SNR *E*_0_/*N*_0_ for data trans.	12 dB~30 dB

Step-size	*μ* = 0.2 and *μ*_max⁡_ = 2

Regularization parameter for ZA-NLMS	*ρ*_ZA_ = 0.0006*σ*_*n*_^2^ for *T* = 1 *ρ*_ZA_ = 0.0002*σ*_*n*_^2^ *L* ^*L*^ ^*L*^ for *T* = 4

Regularization parameter for RZA-NLMS	*ρ*_RZA_ = 0.006*σ*_*n*_^2^ for *T* = 1 *ρ*_RZA_ = 0.002*σ*_*n*_^2^ *L* ^*L*^ ^*L*^ for *T* = 4

Modulation schemes	QPSK, 8PSK, 16PSK, 16QAM, 64QAM, 128QAM
